# Catastrophic STS/SVS type B3 aortic dissection complicated by hepatic portal venous gas and multi-organ malperfusion: a case report

**DOI:** 10.3389/fmed.2026.1836688

**Published:** 2026-06-17

**Authors:** Jia Li, Xinping Lan, Jianbo Guo, Zifang Shang

**Affiliations:** 1 Department of Hepatobiliary Surgery 2, Meizhou People’s Hospital, Meizhou Academy of Medical Sciences, Meizhou, Guangdong, China; 2 Cardiac Intensive Care Unit, Meizhou People’s Hospital, Meizhou Academy of Medical Sciences, Meizhou, Guangdong, China; 3 Department of Imaging, Meizhou People’s Hospital, Meizhou Academy of Medical Sciences, Meizhou, Guangdong, China; 4 Research Experiment Center, Meizhou People’s Hospital, Meizhou Academy of Medical Sciences, Meizhou, Guangdong, China

**Keywords:** aortic dissection, hepatic portal venous gas, malperfusion syndrome, mesenteric ischemia, STS/SVS type B3

## Abstract

**Background:**

Acute aortic dissection (AAD) complicated by visceral malperfusion represents a catastrophic vascular emergency associated with high mortality. The coexistence of hepatic portal venous gas (HPVG) in this setting is an ominous radiological sign, typically indicating irreversible transmural bowel necrosis and breach of the mucosal barrier. We report a fatal case of STS/SVS Type B3 aortic dissection presenting with extensive visceral ischemia and HPVG to highlight the diagnostic significance of this “deadly triad.”

**Case presentation:**

A 64-year-old male with a history of hypertension presented to the emergency department with severe, unremitting abdominal pain of 8 h’ duration. Physical examination revealed significant four-limb blood pressure asymmetry, with a systolic gradient of nearly 100 mmHg between the right and left upper limbs, alongside diffuse abdominal tenderness. Computed tomography angiography (CTA) demonstrated an STS/SVS Type B3 aortic dissection extending from the aortic arch to the iliac arteries. Critical findings included the celiac trunk and superior mesenteric artery (SMA) straddled the dissection flap, representing a typical combination of static and dynamic obstruction. Critical findings included diffuse HPVG, pneumatosis intestinalis, and multi-organ ischemia. Laboratory results showed admission leukocytosis (WBC 27.1 × 10^9^/L) and a massive cytokine storm (IL-6: 14,001.98 pg./mL) with refractory lactic acidosis. Due to the grave prognosis, the family opted for palliative care, and the patient expired 47 h after the decision.

**Conclusion:**

This case underscores the rapid clinical deterioration associated with STS/SVS Type B3 dissection and complex visceral malperfusion. Early CTA recognition of HPVG is vital for identifying irreversible intestinal damage and informing multidisciplinary decision-making in devastating vascular emergencies.

## 1 Introduction

Acute aortic dissection (AAD) is a life-threatening cardiovascular emergency (1). According to the 2022 American Heart Association/American College of Cardiology (AHA/ACC) guidelines, while Stanford type A dissections typically require emergent surgical repair, distal dissections (Stanford type B) are traditionally managed medically unless complicated by rupture or malperfusion syndrome (MPS) (2, 3). However, the newer STS/SVS classification provides a more granular anatomical description. Specifically, a dissection with proximal extension into Zone 3 (STS/SVS Type B3) can compromise the left subclavian artery, leading to upper extremity blood pressure discrepancy, representing a critical clinical sign of complicated cases (4). Beyond arch vessel involvement, the distal extension of the dissection flap can acutely occlude major abdominal branches, resulting in visceral malperfusion, a particularly insidious and catastrophic complication.

When severe visceral malperfusion progresses to transmural bowel necrosis, it often manifests radiologically as hepatic portal venous gas (HPVG). HPVG is not a distinct disease entity but an ominous radiological sign. Recent pathophysiological studies indicate that HPVG typically involves a combination of severe mucosal damage, bowel distension, and the translocation of gas-producing bacteria from the gastrointestinal lumen into the mesenteric venous system (5–7). While HPVG can occasionally arise from benign causes, the latest World Society of Emergency Surgery (WSES) guidelines emphasize that its association with acute mesenteric ischemia almost universally signifies transmural bowel necrosis (8). In this severe context, HPVG serves as a radiological harbinger of impending physiological collapse, carrying a persistent high mortality rate despite modern intensive care advances (9).

The simultaneous clinical presentation of an STS/SVS Type B3 aortic dissection and HPVG is exceptionally rare (10). Recognizing this “deadly triad” of aortic dissection, mesenteric malperfusion, and transmural bowel necrosis requires a high index of clinical suspicion and the timely use of computed tomography angiography (CTA). Herein, we present a rare and fatal case of a 64-year-old male who developed extensive visceral ischemia and HPVG secondary to an STS/SVS Type B3 aortic dissection. By exploring the pathophysiological mechanisms, the dramatic surge of inflammatory biomarkers (such as interleukin-6), and the critical role of early radiological recognition in this specific case, this report aims to heighten clinical awareness and inform multidisciplinary decision-making for this devastating condition.

## 2 Case presentation

A 64-year-old male with a history of hypertension presented to the emergency department after experienced the sudden onset of severe, unremitting abdominal pain. He underwent an initial computed tomography angiography (CTA) at an outside hospital 155 min later, which suggested acute aortic dissection.

On physical examination, the patient was in acute distress. A significant systolic blood pressure discrepancy was noted between the upper limbs (Right: 259/133 mmHg; Left: 162/85 mmHg). This marked gradient was attributed to the intimal flap involving the left subclavian artery origin, leading to severe flow compromise to the left upper extremity. Abdominal examination revealed diffuse tenderness, rebound tenderness, and absent bowel sounds. Notably, bilateral femoral pulses were palpable, suggesting that despite the severe visceral malperfusion, distal limb perfusion remained relatively preserved.

A repeat CTA was performed at our hospital 27 h and 34 min after the initial symptom onset. The images demonstrated an STS/SVS Type B3 aortic dissection extending from the aortic arch to the bilateral external iliac arteries (Figures 1A,B). The primary entry tear, approximately 2.0 cm wide, was located distal to the left subclavian artery. The true lumen was significantly compressed by a dilated false lumen. Further evaluation showed that both the celiac trunk (Figure 1C) and the superior mesenteric artery (SMA; Figure 1D) straddled the dissection flap, resulting in a typical combination of static and dynamic obstruction. The inferior mesenteric artery and bilateral renal arteries originated from the true lumen.

**Figure 1:**
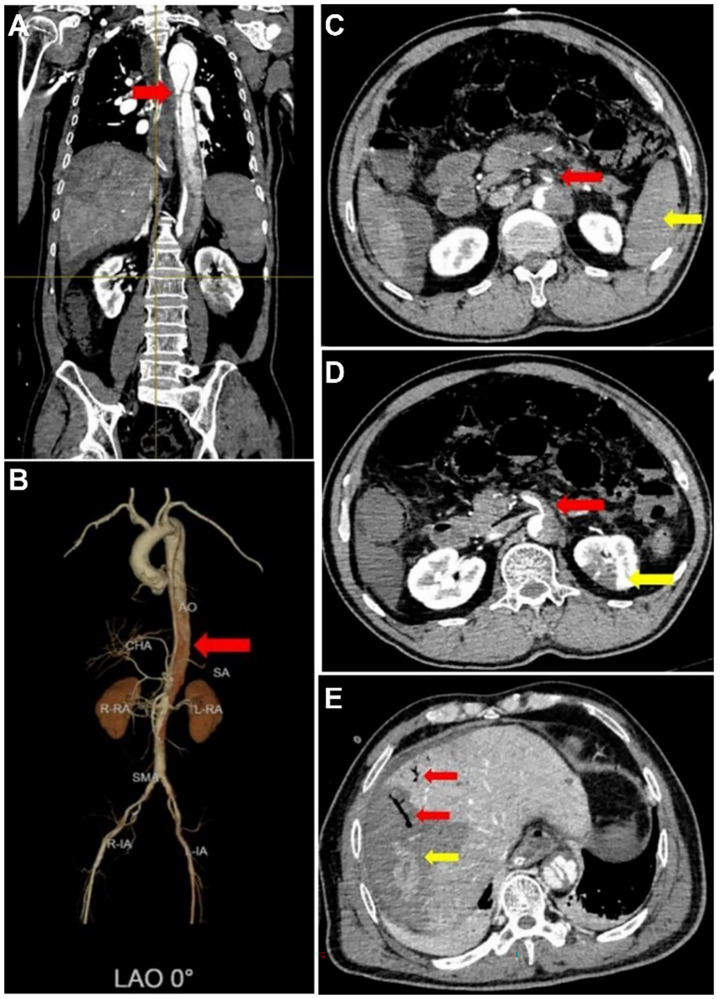
Computed tomography angiography (CTA) of the Stanford type B aortic dissection and its severe abdominal complications. **(A**,**B)** Sagittal and coronal CTA reconstructions demonstrating a Stanford type B aortic dissection. The red arrows indicate the primary entry tear located distal to the left subclavian artery, with a significantly dilated false lumen. **(C)** Axial CT image (arterial phase) at the level of the celiac trunk. The red arrow highlights the celiac trunk originating from the true lumen, which is severely compressed by the high-pressure false lumen, indicating severe dynamic obstruction. The yellow arrow indicates secondary splenic ischemia. **(D)** Axial CT image (arterial phase) at the level of the superior mesenteric artery (SMA). The red arrow points to the dynamic obstruction of the SMA origin due to true lumen compression. The yellow arrow shows partial ischemia of the left kidney. **(E)** Axial contrast-enhanced CT image of the liver. The red arrow clearly demonstrates the classic branching radiolucencies of hepatic portal venous gas (HPVG) extending peripherally to the liver capsule. The yellow arrow indicates patchy areas of hypoperfusion and ischemia in the right hepatic lobe.

Crucially, diffuse gas shadows were identified within the main portal vein and its intrahepatic branches, consistent with HPVG (Figure 1E). The intestinal and gastric walls were thickened with extensive pneumatosis intestinalis. Patchy hypo-enhancement was observed in the right hepatic lobe, pancreas, spleen, and left renal cortex, indicating multi-organ ischemia and early necrosis.

Continuous laboratory monitoring reflected a fulminant systemic inflammatory response and progressive organ failure. As summarized in Table 1, upon admission, the patient exhibited leukocytosis (WBC 27.1 × 10^9^/L). Within 24 h, inflammatory markers soared to extreme levels (e.g., IL-6 reaching 14,001.98 pg./mL), accompanied by severe hepatic and renal impairment, indicating irreversible extensive visceral necrosis. Furthermore, dynamic blood gas analysis (Figure 2) demonstrated a precipitous drop in arterial pH (from 7.33 to 7.01) and persistently rising lactate levels, reflecting severe and refractory metabolic acidosis.

**Table 1:** Key laboratory findings illustrating progressive organ failure.

Parameters	Unit	Admission (Hour 0)	Peak deterioration (Hour 27)	Reference range
White Blood Cells (WBC)	109/L	27.1	20.2	3.5–9.5
Interleukin-6 (IL-6)	pg/mL	-	14,001.98	<7.0
Procalcitonin (PCT)	ng/mL	0.55	2.03	<0.05
D-Dimer	mg/L	2.83	13.89	0.00–1.50
Alanine Aminotransferase (ALT)	U/L	-	355	9–50
Aspartate Aminotransferase (AST)	U/L	-	308	15–40
Creatinine (CREA)	μmol/L	-	250.2	44–97

**Figure 2:**
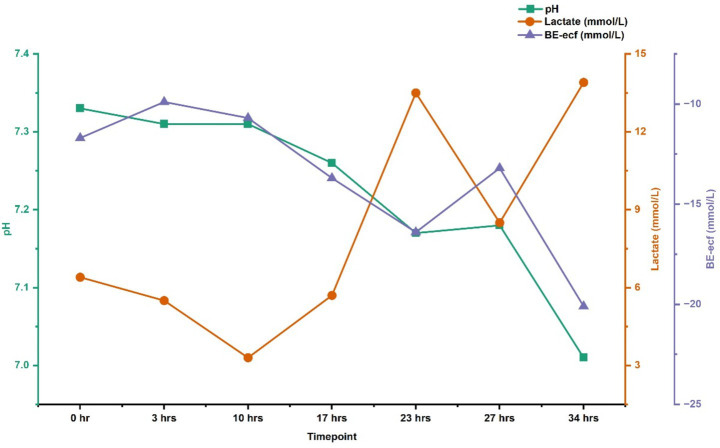
Dynamic changes in blood gas and acid–base status over the 46-h clinical course. The dual-axis line graph illustrates the progressive deterioration of the patient’s metabolic state. The left Y-axis (black/blue line) tracks the precipitous drop in arterial pH, while the right Y-axis (red line) highlights the persistently elevated and surging lactate (Lac) levels, indicative of refractory metabolic acidosis secondary to extensive visceral necrosis.

A multidisciplinary team (MDT) consultation concluded that the patient had extensive visceral necrosis and septic shock. After a detailed discussion regarding the extremely high operative mortality and poor prognosis, the family comprehensively considered the surgical risks and their socioeconomic situation, ultimately opting for supportive palliative care. The patient expired 47 h and 28 min after the decision for conservative management was finalized (which was approximately 55 h after the initial onset of symptoms).

## 3 Discussion

MPS remains one of the most catastrophic complications of AAD, acting as a primary determinant of patient survival. This case is particularly illustrative of the “deadly triad” involving an STS/SVS Type B3 aortic dissection, profound mesenteric malperfusion mediated by both static and dynamic mechanisms, and irreversible transmural bowel necrosis. According to the 2020 STS/SVS reporting standards, the classification of Type B3, indicating proximal extension of the dissection into Zone 3, provides a crucial anatomical explanation for the significant blood pressure discrepancy observed between the patient’s upper limbs (4). While the primary entry tear was situated distal to the left subclavian artery, the retrograde extension of the intimal flap compromised the vessel’s origin, representing a complex variant of distal dissection that necessitates heightened clinical vigilance beyond traditional Stanford categorization (2).

The pathophysiological visceral malperfusion in this instance was driven by a fatal combination of static and dynamic obstruction mechanisms. Dynamic obstruction, characterized by the pressurized false lumen causing the intimal flap to prolapse across branch orifices, was exacerbated by the fixed anatomical distortion of the celiac trunk and SMA straddling the dissection flap (11). This dual-mechanism obstruction ensured a persistent and near-total cessation of blood flow to the midgut and foregut. Such a comprehensive vascular compromise explains the exceptionally rapid progression from the initial onset of pain to the development of hepatic portal venous gas (HPVG). While HPVG can occasionally arise from benign etiologies, the latest World Society of Emergency Surgery (WSES) guidelines emphasize that in the context of acute mesenteric ischemia, its presence almost universally signifies irreversible intestinal gangrene and a breach of the mucosal barrier (8, 12).

The laboratory trajectory observed in this case offers a high-resolution window into the resulting physiological collapse. The early presentation of leukocytosis (WBC 27.1 × 10^9^/L) served as an initial biomarker of severe tissue stress and the incipient translocation of gut-derived pathogens. This progressed rapidly into a massive cytokine storm, with interleukin-6 (IL-6) levels peaking at 14,001.98 pg./mL. Recent pathophysiological studies confirm that such extreme elevations in inflammatory mediators, alongside the refractory lactic acidosis and precipitous pH drop observed in our patient, reflect a state of systemic inflammatory response syndrome (SIRS) that typically precedes multi-organ failure (13, 14). In such advanced stages, as demonstrated by the extensive pneumatosis intestinalis and HPVG on CTA, the window for successful surgical salvage often closes, underscoring the critical need for early radiological recognition and rapid multidisciplinary intervention before the onset of irreversible metabolic collapse.

Given this devastating systemic collapse, the management of AD complicated by severe mesenteric ischemia remains a profound clinical dilemma. The traditional treatment paradigm involves immediate endovascular interventions (such as TEVAR) to restore true lumen flow, potentially followed by exploratory laparotomy for necrotic bowel resection. However, when HPVG and septic shock are already established, the patient’s physiological reserve is utterly depleted. In this case, the multidisciplinary team (MDT) concluded that the patient could not tolerate the physiological insult of massive bowel resection and simultaneous aortic repair, rendering conservative, palliative care the only humane option.

Limitations of this case report include the lack of histopathological confirmation (autopsy) of the necrotic organs, as the diagnosis was established strictly on clinical and radiological grounds. Furthermore, as a single case, it highlights a grim natural history but cannot offer comparative data on the efficacy of earlier interventions.

## 4 Conclusion

The coexistence of aortic dissection and HPVG constitutes a “deadly triad” of vascular tear, malperfusion, and transmural bowel necrosis. HPVG serves as an ultra-high-risk radiological harbinger of irreversible physiological collapse. This case emphasizes that when handling acute abdominal pain with asymmetric blood pressure, prompt CTA is paramount. Radiologists must rapidly identify not only the intimal flap but also distal branch vessel compromise and subtle signs like HPVG. Immediate multidisciplinary assessment is crucial to determine if emergent revascularization is feasible before the onset of the irreversible cytokine storm and septic shock.

## Data Availability

The raw data supporting the conclusions of this article will be made available by the authors, without undue reservation.
